# Metal-Assisted Catalytic Etching (MACE) for Nanofabrication of Semiconductor Powders

**DOI:** 10.3390/mi12070776

**Published:** 2021-06-30

**Authors:** Kurt W. Kolasinski

**Affiliations:** Department of Chemistry, West Chester University, West Chester, PA 19383, USA; kkolasinski@wcupa.edu; Tel.: +1-610-436-2968

**Keywords:** metal-assisted etching, metal-assisted catalytic etching, silicon nanowires, porous silicon, porous powders, metal nanoparticles, deposition

## Abstract

Electroless etching of semiconductors has been elevated to an advanced micromachining process by the addition of a structured metal catalyst. Patterning of the catalyst by lithographic techniques facilitated the patterning of crystalline and polycrystalline wafer substrates. Galvanic deposition of metals on semiconductors has a natural tendency to produce nanoparticles rather than flat uniform films. This characteristic makes possible the etching of wafers and particles with arbitrary shape and size. While it has been widely recognized that spontaneous deposition of metal nanoparticles can be used in connection with etching to porosify wafers, it is also possible to produced nanostructured powders. Metal-assisted catalytic etching (MACE) can be controlled to produce (1) etch track pores with shapes and sizes closely related to the shape and size of the metal nanoparticle, (2) hierarchically porosified substrates exhibiting combinations of large etch track pores and mesopores, and (3) nanowires with either solid or mesoporous cores. This review discussed the mechanisms of porosification, processing advances, and the properties of the etch product with special emphasis on the etching of silicon powders.

## 1. Introduction

It has long been known that metals can spontaneously deposit on semiconductors out of solutions containing dissolved ions [1,2,3]. The kinetics of this process are complex, only partially understood, and depend on the concentration of the metal ion as well as the available counter reaction [4,5]. Thermodynamically, at a standard concentration with the evolution of H_2_ as a counter reaction, any metal with a positive reduction potential, for example, W, Re, Bi, Cu, Po, Ru, Hg, Ag, Au, Pd, Pt, Rh, Ir, and Tl, can deposit spontaneously on a semiconductor. When coupled to a sufficiently exergonic counterreaction such as the formation of a stable oxide, even metals with a slightly negative reduction potential might be induced to deposit, though deposition would be limited if the oxide is insulating. Any metal that has a standard reduction potential less positive than the semiconductor’s valence band maximum (VBM) exhibits kinetically constrained deposition. Specifically for Si, the metals W, Re, Bi, Cu, Po, and Ru that have *E*° < 0.6 V have slow kinetics for deposition of the metal. These metals as well as metals with a negative *E*° are also susceptible to rapid dissolution in the presence of oxidants such as NO_3_^−^ or H_2_O_2_ in solution. In contrast, the metals Hg, Ag, Au, Pd, Pt, Rh, Ir, and Tl that have *E*° > 0.6 V have facile kinetics for metal deposition onto Si particles. Substantial differences in the kinetics of Cu deposition as compared with Ag, Au, and Pt have been verified experimentally by Schönekerl and Acker [4].

If the reduction potential of a metal ion is more positive than the VBM (both expressed in equivalent units), deposition proceeds without a substantial kinetic barrier. The kinetics of deposition depend on the relative energies of the metal ion acceptor level as compared to the VBM, and also depend on the overlapping density of states. Because the density of states at the Fermi energy of a metal is orders of magnitude higher than the density of states at the VBM, the kinetics of electron transfer inherently favor deposition of metal onto metal rather than onto the bare semiconductor. Metal atoms tend to be strongly bound to semiconductor surfaces; nonetheless, their lateral potentials are rather flat and exhibit low diffusion barriers. Furthermore, metal atoms tend to interact more strongly with similar atoms rather than atoms in the semiconductor. The combination of these three factors means that metals that spontaneously deposit onto semiconductors have a tendency to deposit as nanoparticles (Volmer–Weber growth) rather than as uniform layers (van der Merwe growth) [6].

An enhanced rate of electron transfer in metals is true for other metals and any oxidant. When a semiconductor surface that is decorated with a partial covering of metal nanoparticles is exposed to an oxidant in solution, the oxidant preferentially accepts an electron from the metal [7]. In addition, metals can act electrocatalytically. Consequently, for an oxidant such as H_2_O_2_ or bubbled O_2_ [8,9,10,11], reduction occurs almost exclusively at the metal nanoparticles, and one should expect that electron transfer at a metal-covered semiconductor surface is highly anisotropic. Interestingly, Hu, et al. [12] found that the rate of etching by dissolved O_2_ was increased significantly if Si with a patterned deposit of Ag, Au, or Pt was attached to a graphite substrate.

Early investigations of the pitting and etching of Si in the presence of metals has been well detailed by Huo et al. [13]. These studies were performed in conjunction with the need to suppress metal impurities on the surfaces of Si wafers in order to obtain reliable performance in ultra-large-scale integration (ULSI) devices. Li and Bohn [14] demonstrated that thin Au, Pt, or Au/Pd layers could be used to localize the formation of photoluminescent porous silicon (por-Si) in a manner closely related to the galvanic etching [15] process discovered by Kelly et al. [16,17]. However, it was the discovery by KQ Peng et al. [13,18] that such anisotropic electron transfer could be used to initiate preferential directional etching of silicon that transformed metal-assisted catalytic etching (MACE, also known as metal-assisted etching (MAE), metal-assisted chemical etching, and MacEtching) into a technique for controlled micro- and nano-structuring beyond porosification. Micromachining with MACE is possible because of the formation of etch track pores of roughly the same size as the diameter of the metal nanoparticle. These etch tracks can either be straight, as first observed in the early work of Peng et al., or helical, as was observed by Tsujino and Matsumura [19]. In a modification on this scheme, Azeredo et al. [20,21] and Bastide et al. [22] developed systems for pattern transfer based on patterned metal stamps. A more distantly related micromachining technique utilized a metal needle electrode to anodically bore through-holes in Si [23].

Peng et al. [24] and Fang et al. [25] recognized that large, ordered arrays of etch tracks could be formed through control of the structure of the deposited metal. Building on techniques known as natural [26], nanosphere [27], colloidal [28,29] or hole-mask colloidal lithography [30], close-packed [31] or non-close-packed arrays [32] of polystyrene or silica nanospheres or Ni islands [33] were self-assembled to serve as masks to form ordered arrays of either metal nanoparticles or holes in metal layers. The formation of etch track pores from such structured metal layers, thus, led to the formation of either ordered arrays of pores, when the metal nanoparticles were aligned, or ordered arrays of silicon nanowires (SiNW), when the holes were aligned. Control over the tapering of SiNWs has been demonstrated [34] and improved upon by changing the H_2_O_2_ concentration or using an applied bias [35,36].

These lithographic techniques can be implemented to machine single-crystal wafers of silicon [13,37,38] as well as other semiconductors [29,39,40]. The advantageous properties of MacEtched structures have made them ripe for exploitation in a wide variety of applications [40,41,42]. Surface-enhanced Raman spectroscopy (SERS) was performed with electric field enhancement being provided by the same metal nanoparticles used to catalyze MACE [43]. Although the residual presence of metal impurities can be deleterious for many electronic and optoelectronic applications, micromachining with MACE has been used to investigate the production of materials for a variety of applications including optical sensors [44] and photovoltaics [39]. Under the proper conditions, MACE has readily led to the formation of low-reflectivity black silicon [36,38,45], and the effect was enhanced by MACEing a pyramid-covered Si surface [46]. This low reflectivity has been used to improve the efficiency of solar cells [47]. Ag-catalyzed MACE has also been used to produce photoluminescent hierarchical porous layers, for which the wavelength of photoluminescence (PL) was tuned in response to the concentration of H_2_O_2_ used as an oxidant [48]. Photoluminescence of SiNWs was found to be sensitive to exposure to O_2_ [49], and PL quenching of porous SiNWs was used to demonstrate a reversible O_2_ sensor [50].

Silicon nanostructures, both por-Si and SiNWs, are of great interest in multiple energy applications. Nanostructures formed with MACE have been investigated for energy harvesting applications such as thermoelectric [51] and piezoelectric generators [40]. Extensive activity [40] has been directed toward high-performance Li-ion battery (LIB) anodes using either SiNWs [52,53,54] or por-Si [55,56].

The surface chemistry [57,58,59,60,61] and structure of etched Si is extremely versatile. SiNWs produced by MACE can be either solid core or porous [62] depending largely on the resistivity of the Si substrate. Low-doped Si, regardless of whether it is n type or p type, leads to solid core SiNWs. Highly doped Si succumbs to remote etching and the formation of porous SiNW [36,63,64,65,66]. SiNWs have been modified with fluoroalkylsilanes to exhibit superhydrophobicity [67], and their rate of biodegradability was regulated by controlling their termination [64]. MACE has also been applied to a number of areas of biomedicine [68] such as the production of microneedles [63], for delivery of, for example, the anticancer drug doxorubicin [69], and the generation of biocompatible nanoparticles [70].

Applications that require macroscale structures such as microfluidics, and microelectromechanical systems (MEMS) [40] have also been pursued. If metal rather than Si nanowires is desired, the metal nanoparticle at the bottom of the etch track pore can be used as a catalyst for metal deposition within the pores. Subsequent removal of the Si pore walls leaves free-standing metal nanowires [71].

The use of single-crystal substrates is extremely useful in scientific studies because of their well-defined impurity levels, crystallographic orientation, and planar structure. Single-crystal substrates are also advantageous for some applications such as MEMS and microfluidics because etch track pore formation is highly directional in nature [72,73]. For wafers, lithographic and growth methods developed primarily for the electronics industry can be exploited and combined with MACE to generate devices that integrate a range of electronic, optoelectronic, optical, and transport properties. This can be particularly useful in sensor, photovoltaic, or energy conversion applications. However, single-crystal wafers are extremely expensive, especially for any material other than silicon. In pharmaceutical applications, where purity is of utmost concern and cost is essentially of no concern, wafers can represent a viable option. However, for bulk production of nanowires or in applications such as LIB anodes, consumer products, and water filters, the cost of wafers is prohibitive. These applications necessitate the use of powdered semiconductors rather than wafers. While the vast majority of MACE studies and reviews have concentrated on wafers [13,37,39,40,68,74,75,76,77], the focus of this review will be on the etching of powders.

## 2. Mechanistic Aspects of MACE

The development of a mechanistic understanding of MACE has been greatly informed by the recognition of the similarities between the Si electroless etching schemes known as stain etching [78], regenerative electroless etching (ReEtching) [79], and MACE [7,72]. In stain etching, an oxidant injects a hole into the silicon valence band to initiate reaction. While nitrate (usually introduced from nitric acid [80]) is the most commonly employed oxidant, the extremely complex mechanism of nitrate reduction [81,82,83], complete with multiple intermediates and various gaseous products [84], makes the formation of homogeneous and thick porous films challenging. The discovery [85,86] that an oxovanadium(V) species is optimally coupled to the Si valence band greatly enhanced the utility of stain etching, which is characterized by the following overall chemical reaction.

6HF(aq) + Si(s) + 2VO_2_^+^(aq) → SiF_6_^2−^(aq) + 2VO^2+^(aq) + 2H_2_O(l) + H_2_(g), *E*° = 2.231 V (1)

Note the large standard reduction potential, *E*°, of this reaction, which should push the reaction to completion except for the coupling of the reaction rate to the silicon band structure through the effects of quantum confinement [87,88]. More importantly for the optimization of electroless etching, the interconversion of oxovanadium ions between the +5 and +4 oxidation states in hydrofluoric acid is completely reversible in the presence of Si and H_2_O_2_ [89]. Regeneration of the +5 state is required to initiate Rxn. (1) and is affected by Rxn. (2) as follows:

H_2_O_2_ + 2VO^2+^ → 2VO_2_^+^ + 2H^+^, *E*° = 0.785 V (2)

**Figure 1:**
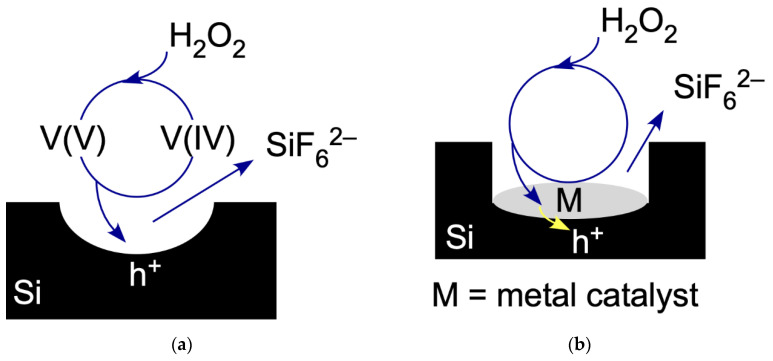
(**a**) The ReEtch cycle for etching of Si. An oxovanadium ion with vanadium in +5 oxidation state, denoted V(V), injects a hole, h^+^, in the Si valence band and is reduced to a vanadium ion in +4 oxidation state, V(IV). The hole initiates etching of Si atoms to form a pore in the substrate. The product of the complete reaction is SiF_6_^2−^. Injected H_2_O_2_ removes an electron from V(IV) to regenerate V(V) so that the cycle can begin again. (**b**) The injection MACE cycle for etching Si, denoted iMACE. Injected H_2_O_2_ removes an electron from a metal nanoparticle, M, which then injects a hole into the Si substrate. The hole initiates the etching of Si forming the etch product SiF_6_^2−^, to form a pore in the substrate.

As illustrated in Figure 1a, this reversibility allows us to establish an etching cycle. In this cycle, VO_2_^+^ acts as a catalyst that facilitates the porosification of Si by H_2_O_2_ according to the following reaction:

(3) $$6\mathrm{HF}(\mathrm{aq})+\mathrm{Si}(s)+H_{2}O_{2}(\mathrm{aq})\;\overset{\;VO_{2}^{+}\;}{\to }\;H_{2}{\mathrm{SiF}}_{6}{}^{2-}(\mathrm{aq})+2H_{2}O(l)+H_{2}(g),\;E{}^{\circ}=3.016\;V$$

This is quite remarkable because Si acts as a very poor catalyst for H_2_O_2_ decomposition, which means that the etch rate of Si in HF + H_2_O_2_ solutions in the absence of a catalyst is extremely low [90]. The introduction of H_2_O_2_ as an oxidant is advantageous because it is inexpensive and widely available, and also it can be introduced with a syringe pump (details in the next section) to control the rate and extent of reaction [89].

Figure 1b illustrates that we can heterogenized the catalyst by replacing VO_2_^+^ with a deposited metal M. In other words, the overall electrochemical reaction governing MACE is as follows:

(4) $$6\mathrm{HF}(\mathrm{aq})+\mathrm{Si}(s)+H_{2}O_{2}(\mathrm{aq})\;\overset{\;M\;}{\to }\;H_{2}{\mathrm{SiF}}_{6}{}^{2-}(\mathrm{aq})+2H_{2}O(l)+H_{2}(g),\;E{}^{\circ}=3.016\;V$$

As discussed in [7], there are three possible etch pathways: (1) divalent dissolution requiring the injection of two holes per Si atom etched, (2) tetravalent valent dissolution requiring the injection of three holes per Si atom etched that does not involve an SiO_2_ intermediate, and (3) tetravalent dissolution that requires an SiO_2_ intermediate. Divalent dissolution is accompanied by H_2_ formation. The tetravalent pathways are both not accompanied by H_2_ formation. Equation (4) implies divalent dissolution of Si. Divalent dissolution was directly observed for Ag- and Au-catalyzed MACE when V_2_O_5_ was used as the oxidant [7]. The same study determined that Pt-catalyzed MACE followed a tetravalent pathway and Pd-catalyzed MACE followed a mixture of di- and tetra-valent dissolution. Unfortunately, the stoichiometry of H_2_O_2_-induced etching could not be determined in that study. Wang et al. [64] detected but did not quantify the amount of H_2_ formed by Ag-catalyzed MACE with H_2_O_2_ as oxidant. This meant that either divalent or a mixture of divalent and tetravalent dissolution was occurring in this system. When etching is divalent, formation of SiO_2_ and its removal by HF etching is not an important reaction pathway. Similarly, since H_2_ is not formed in tetravalent electropolishing via SiO_2_ formation, the detection of H_2_ during Ag-catalyzed MACE with H_2_O_2_ means that divalent dissolution must be occurring (though perhaps not exclusively).

All the processing advantages concomitant with H_2_O_2_ injection can also be realized in metal-assisted etching, a process that can be recast as injection-MACE (iMACE). The advantages of iMACE lie in the ability to control the extent and rate of etching by controlling the volume and rate, respectively, of H_2_O_2_ injection; and also, in the establishment of steady-state etching conditions in which the concentration of oxidant is minimal. The introduction of injection enabled quantitative studies of MACE [66] in which it was discovered that the nature of the etching process depended on the loading of the deposited metal. Furthermore, Tamarov et al. [65] found that because of the low oxidant concentration, Cu could be used as a catalyst to porosify Si, not just anisotropically etch it.

Whereas there are analogies in the initiation and the overall electrochemistry of electroless etching schemes, there are also important differences. The obvious difference between ReEtching and MACE, represented in Figure 1, is the presence of the metal catalyst in contact with the Si surface. The presence of the metal and the directing of catalytic activity toward specific locations gives rise to several issues that can be traced back to electron transfer at the solution/metal interface and hole injection at the metal/semiconductor interface:

1. Etching can be either local or remote.
2. Local etching results in the formation of etch track pores that have a width and shape determined by the size and shape of the metal deposit.
3. Remote etching generates tortuous pores analogous to ReEtching.
4. The space charge layers (SCL) around metal nanoparticles interact with one another, which facilitates co-operative etching.
5. The efficacy of hole injection at the metal/semiconductor interface depends on the elemental composition of the metal, semiconductor doping, and the temperature.
6. The balance between remote and local etching can be controlled by metal nanoparticle size, metal nanoparticle density, elemental composition of the metal, semiconductor doping, and temperature; therefore, these parameters are used to control the pore size distribution as well as whether the walls of etch track pores are solid or porous.

**Figure 2:**
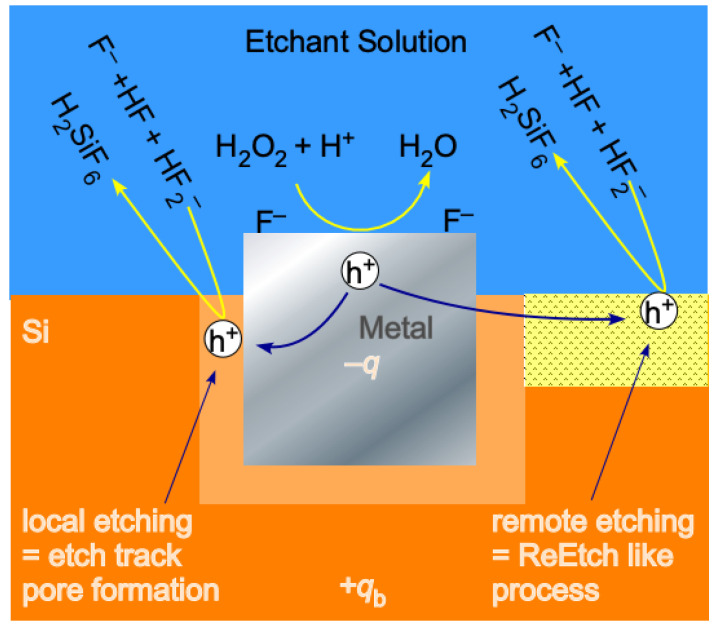
Hole are generated by the decomposition of H_2_O_2_ catalyzed by reaction on the surface of metal nanoparticles. Holes trapped at the metal/semiconductor interface initiate local etching and etch track pore formation. Holes that escape the space charge layer initiate remote etching which generates tortuous pores similar to those generated by ReEtching.

These six points are illustrated schematically in Figure 2 and are demonstrated quantitatively in the Results section. In Figure 2, we see that holes are generated by electron transfer to the oxidant on the surface of the metal nanoparticle catalyst. The nature of band bending at the metal/semiconductor interface [35,36,65,91,92] is determined by the alignment of the metal Fermi level with the semiconductor VBM. Therefore, both the height (and whether the contact is Ohmic or Schottky-like in nature) and width of the barrier that impedes transport of the hole away from the metal/semiconductor interface depends on the composition of the metal and the doping of the semiconductor. In particular, the width of the SCL narrows at high doping levels (regardless of the type). This facilitates the escape of holes by tunneling through the Schottky barrier and into the remote region. This behavior explains, as first observed by Hochbaum et al. [62], why highly doped samples etch to form mesoporous pore walls surrounding etch track pores.

Another way to conceptualize this system is in terms of fields and chemical potential. The idea that band bending and charge imbalance on the metal nanoparticle polarizes the surrounding Si to engender local electropolishing beneath the metal nanoparticle and remote porosification away from the metal nanoparticle was first proposed and supported by band bending calculations by Kolasinski in 2014 [93], then, confirmed through quantitative two-dimensional band bending modelling by Torralba et al. in 2016 [35], and confirmed again by Wang et al. in 2018 [64]. Tamarov et al. [65] expanded these concepts by interpretation of extensive experimental data through theoretical calculations for a full range of heavily, moderately, and lightly doped p and n type Si in combination with Cu, Ag, Au, Pd, and Pt catalysts.

Rezvani et al. [94] directly measured that the metal nanoparticles are biased negative with respect to Si during MACE. One consequence of this effective negative charge is the formation of an image dipole that attracts the metal to the semiconductor. This is important to explain [72] the etching of particles on all sides for which, unlike the illustration shown in Figure 2, there are no natural up or down directions. A second consequence is that the metal nanoparticle effectively acts as a local potentiostat powered by H_2_O_2_ decomposition that polarizes the surrounding Si. In this picture, the local region is biased into the electropolishing regime, which completely removes Si and forms etch track pores beneath the metal catalyst. Further away in the remote region, the Si is biased below the critical potential and the semiconductor etches in the mesopore formation regime.

After hole transport to the solution/semiconductor interface, solution-phase species such as F^−^, HF, and HF_2_^−^ are involved in the various steps of the reaction mechanism [95]. These species move under the influence of the electrochemical potential. As demonstrated by Equation (4), there is a huge driving force for these species to react with Si to form H_2_SiF_6_(aq). However, without a source of holes, the reactants are kinetically constrained and the etch rate is minimal; only with the push of the oxidant and the pull of the fluoride species, can the activation barrier be overcome to complete this full electrochemical circuit.

## 3. Materials and Methods

Wafers represent a well-defined, low-surface-area substrate. The etching of powders presents numerous challenges that are not obvious because they are not of much relevance to wafer etching. Wafer etching is usually performed under the tacit assumption of an excess of etchant and the influence of concentration changes or reactant depletion are generally not considered. Few of the multitude of studies addressing the etching of wafers ever report the volume of etchant used or the extent of reaction. Rarely is the temperature controlled. Therefore, from most of the published data, no reasonable inferences can be made regarding the effects of changes in concentration and temperature during etching. When etching is extensive enough to generate an easily weighable mass difference, then, reactant depletion must be taken into consideration. Two other factors, discussed further by Tamarov et al. [65] that complicate quantitative analysis are that etching and Si mass loss occur during metal deposition and the presence of side reactions of the oxidant. It should also be noted that when pores become long, transport constraints hinder uniform mixing as is required for simple application of a rate law. Extrapolation to zero film thickness may be required to extract kinetically relevant data [96,97].

The low specific surface area of wafers greatly reduces the absolute rate of reaction as compared with powder etching. This translates into much reduced gas and heat production. Gas production is important because the formation of bubbles [98] can generate capillary forces, which can engender structural changes much like those observed during drying. SiNWs are susceptible to bunching under the influence of mutual van der Waals forces [99]. Bunching was exploited by [44] to create hot spots for electromagnetic field enhancement that improves the sensitivity of SERS analysis. The surface tension of a drying solvent plays a role in nanowire bunching [100]. The magnitude of capillary forces in nanostructured systems can far exceed van der Waals forces. Just as they can lead to cracking and various structural transitions in drying colloidal [101] and porous films [102,103], capillary forces can readily cleave the walls between etch track pores. As we shall see in the next section, this is the method by which nanowires are formed in MACE of powders [72]. Understanding the roles of mutual attraction and capillary collapse along with the introduction of electrostatic repulsion has facilitated the creation of SiNWs with exceptionally high aspect ratios [104].

Another problematic aspect of gas bubble formation is that it can make silicon buoyant. For a wafer, the troublesome floating of a sample to the surface of the etchant is easily overcome by securing the sample in a fixed position. A vertical orientation should be chosen so as to avoid trapping of gas bubbles under the sample. In a vertical configuration, the bubbles have a beneficial effect by ensuring thorough mixing in the solution. Overly vigorous bubbling can lead to structural changes and exfoliation. For powders, buoyancy results in foaming, which is extremely deleterious to the etching process because it causes inhomogeneous and incomplete etching as well as the possibility of overtopping reaction vessels.

Thermal management has not been recognized as a concern for MACE of wafers even though it is known that the etch rate exhibits Arrhenius behavior with an activation energy of ~0.4 eV [72,105]. However, the standard reduction potential, *E*°, of the overall etching reaction (4) is 3.016 V which translates into a molar Gibbs energy of reaction of Δ_r_*G*_m_° = −1164 kJ mol^−1^ as compared with the molar Gibbs energy change for Si combustion to SiO_2_ which is only −856 kJ mol^−1^. This means that etching is extremely exothermic. For the etching of powders this can translate into temperature rises that are sufficient to boil the etchant and fully oxidize the product to SiO_2_. It is essential during the etching of powders that heat dissipation is built into the design of the reaction vessel.

Agglomeration of particles is problematic for powder etching. While this is of no concern during etching, it does arise during metal deposition, as discussed later. In the etching of wafers, HF has been replaced with NH_4_F. This allowed Gonchar et al. [106] to vary the length and shape of etched structures. However, the precipitation of salts or hexafluorosilicates out of solution has been observed during porous silicon formation [107]. The effects can be deleterious for homogeneity and continuous etching. On wafers, precipitation is easily suppressed by avoidance of cations that might promote precipitation; however, when etching powders essentially to completion and in large batches, avoidance of added ammonium, alkali, or alkaline earth cations is even more critical.

The exigencies of etching powders—exercising as much control and uniformity as possible with a highly exothermic reaction involving three phases—mean that commonplace methods derived from wafer cleaning procedures are of little use. It is totally insufficient to simply mix reagents in the required proportions and either dip the sample in the etchant or pour the etchant onto the sample followed by rinsing and drying.

When anodization is used to produce por-Si films, ethanol is commonly used as a surfactant to minimize bubble formation, ease the release of H_2_ bubbles from the substrate, and enhance homogeneity [108,109]. Ethanol is not a wise choice for conventional stain etching because strong oxidants such as nitric acid can react violently with ethanol. Ethanol has been shown to significantly decrease the rate of stain etching with V_2_O_5_ as the oxidant [96]. Ethanol is slowly oxidized by V_2_O_5_ dissolved in HF(aq); therefore, etchants need to be made up fresh and cannot be stored. At low concentration, ethanol-hydrogen peroxide mixtures are stable at room temperature; nonetheless, a much better choice of surfactant is acetic acid, which is much less reactive because it is the partial oxidation product of ethanol. While it is found to decrease the etch rate, the use of acetic acid greatly reduces foaming during stain etching or ReEtching [110]. Foaming is also suppressed by the use of larger particles, such that the concentration of acetic acid required to ameliorate foaming decreases with increasing particle size.

In ReEtching, Si powder is first dispersed in acetic acid appropriately diluted with deionized water. ReEtching can be performed on particles of any shape or size. While the initial report [79] demonstrated complete etching through the core for 4 µm particles, subsequent work in our lab has found that particles at least as large as 50 µm can be completely etched. Etching must be performed is a fluoropolymer, polyethylene (either high-density HDPE or low-density LDPE) or polypropylene container because of the use of hydrofluoric acid. A magnetic stirring bar is used for agitation. Continuous sparging with N_2_ is sometimes used to aid in agitation, suppression of foaming, and exclusion of O_2_. The N_2_ stream can also be cooled; however, it is essential to place the reaction container in a thermostatic water (or ice/water) bath to ensure sufficient cooling and temperature regulation. Other cooling systems have also been employed [111].

V_2_O_5_ dissolves readily in room temperature concentrated HF(aq) but extremely slowly in chilled or dilute HF. Therefore, V_2_O_5_ is added directly to concentrated HF(aq) in a chemically resistant polymer container before dilution and cooling. After dissolution and cooling, the dissolved V_2_O_5_ is added to the dispersed Si. In the absence of acetic acid, the addition of HF to Si powder dispersed in water causes the formation of a silvery film on top of the solution because HF reacts with the native oxide layer on the Si particles and replaces hydrophilic Si–OH groups with hydrophobic Si–H groups. Such a layer forms whenever particles under ~100 µm are present in the dispersion and has an appearance much like that of an unpolished Si wafer.

The etchant with dispersed Si powder is allowed to equilibrate under constant stirring. Then, H_2_O_2_ is introduced with a syringe pump at a rate sufficient to deliver the desired amount of oxidant over a period of typically 30–90 min. In conventional MACE of wafers, all the oxidant is added at the beginning of the etch. The etch rate depends on the concentration of H_2_O_2_ [48,64]. When injection is used to deliver the oxidant, a low steady-state concentration is maintained that is set by a number of factors including the volume of the solution, the concentrations of silicon and oxidant, and the injection rate. For both ReEtching [79] and MACE [65,66], characteristics such as the yield of the reaction, extent of reaction, and thickness of the layer depend on the etching time/injection rate and the H_2_O_2_ to Si molar ratio.

Subsequent to etching, the silicon must be rinsed and filtered to remove the etchant and etch products. Details of the rinsing protocol are important for obtaining a high-quality product. The structural integrity of the etched film is strongly affected by the capillary forces that arise during solvent drying. Retention of visible photoluminescence after air exposure is also strongly dependent on the rinsing protocol [112]. Simple rinsing with deionized water is by far the least acceptable procedure. Rinsing with dilute HCl(aq) is superior for removing etchant and etch products; however, the high surface tension of water leads to significant structural damage and the introduction of nonradiative traps. Thorough rinsing with dilute acid to remove the bulk of the etchant should be followed with rinsing with ethanol, and then wetting with pentane, followed by vacuum oven drying. Skipping the ethanol rinse has only a moderate effect on PL retention but wetting with pentane should be performed.

The abovementioned procedures apply equally well for ReEtching and MACE, except for two issues. Obviously, no dissolved V_2_O_5_ is required for MACE. More importantly, the rinsing protocol must be changed. For MACE, dilute HCl(aq) must be avoided to preclude the precipitation of metal chloride into the etched powder. Sometimes it is desirable to remove the metal from the Si powder. This should be performed before the pentane wetting step. In addition, to avoid damage to the Si nanostructures, a metal such as Ag should be removed by concentrated HNO_3_ that has been diluted with concentrated acetic acid that has been chilled [66].

MACE requires a metal deposition step prior to introduction of the oxidant, which also can be performed on powder particles of arbitrary shape, size, or doping. Control of the uniformity and amount of deposited metal is extremely important [65,66]. The conventional high-load MACE regime (HL-MACE) is obtained when metal is deposited at the level of 1 mmol per g of Si. The more recently discovered low-load MACE regime (LL-MACE), in which the porosity and pore size distribution are scalable, is obtained when metal is deposited at the level of <0.05 mmol per g of Si. In order to enhance uniformity, avoid agglomeration, reduce the strength of agglomeration, and precisely control the amount of metal deposited, the silicon powder is again dispersed in acetic acid, HF is added, and the dispersion is cooled in a thermostatic bath with constant agitation in the form of magnetic stirring. Then, the dissolved metal salt is injected with a syringe pump over a period of typically 15–20 min. Subsequent to metal deposition, injection of the oxidant is performed, as described above, directly into the same solution that was used for deposition.

## 4. Results of Powder Etching

**Figure 3:**
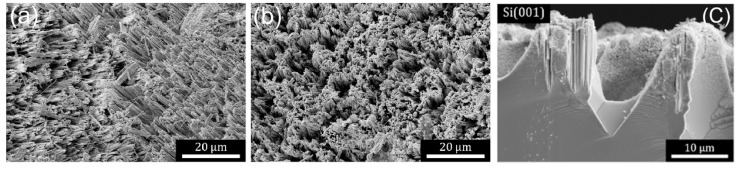
Secondary electron scanning electron microscope (SEM) images of etched Si particles using Ag-catalyzed HL-MACE. (**a**) Single crystal and (**b**) metallurgical-grade Si powders both etch to reveal primarily parallel etch track pores as a result of etching with HL-MACE. (**c**) Etching of a textured Si(001)-oriented wafer shows that the Ag nanoparticles etch primarily along {001} directions irrespective of the incline of the external surface of the substrate. Copyright © 2021 Kolasinski, Unger, Ernst and Aindow. Reproduced under Creative Commons Attribution License (CC BY) from Ref. [72].

Using the techniques described in the previous section, electronics grade (EG-Si) or metallurgical grade (MG-Si) silicon with arbitrary size and characteristics can be etched: single crystalline or polycrystalline, n or p type, highly doped/impure, or low doped. Figure 3 demonstrates that both EG- and MG-Si powder particles are etched by conventional HL-MACE to generate particles with roughly the same morphology; numerous, mostly parallel etch track pores are formed leaving behind random ridge-like structures [72]. The formation of etch track pores primarily directed along {001} directions is confirmed by cross-sectional scanning electron microscope (SEM) images from both powders and single-crystal wafers, as shown for the latter in Figure 3c. The structure of the particles shown in Figure 3 is consistent with SEM images reported by Ouertani et al. [113], who also found that Ag-catalyzed MACE of MG-Si tends to improve the crystallinity due to the removal of amorphous material and impurities. The removal of impurities resulting from MACE to upgrade MG-Si to solar-grade Si has been studied extensively by Li et al. [114,115,116].

The advantage of being able to process particles of arbitrary size, shape, and doping has been further demonstrated by Kozlov et al. [70]. They have shown that centimeter-size particles of MG-Si, potentially even particles reclaimed from another process, can be subjected to Ag-catalyzed MACE and subsequently pulverized with sonication in water to generate photoluminescence por-Si nanoparticles.

Differences in the results of etching in the HL regime depending on the metal have been reported, for example, by Matsumoto et al. [117] for Ag, Au, and Pt. Pinna et al. [36] performed extensive experiments of Ag-catalyzed HL-MACE to determine the effects of changing doping levels and types. The doping level is important because it affects the balance between local etching in the vicinity of the metal nanoparticle (favored at low doping) versus remote etching that forms tortuous mesopores (which occurs at high doping) [36,64,65,66].

**Figure 4:**
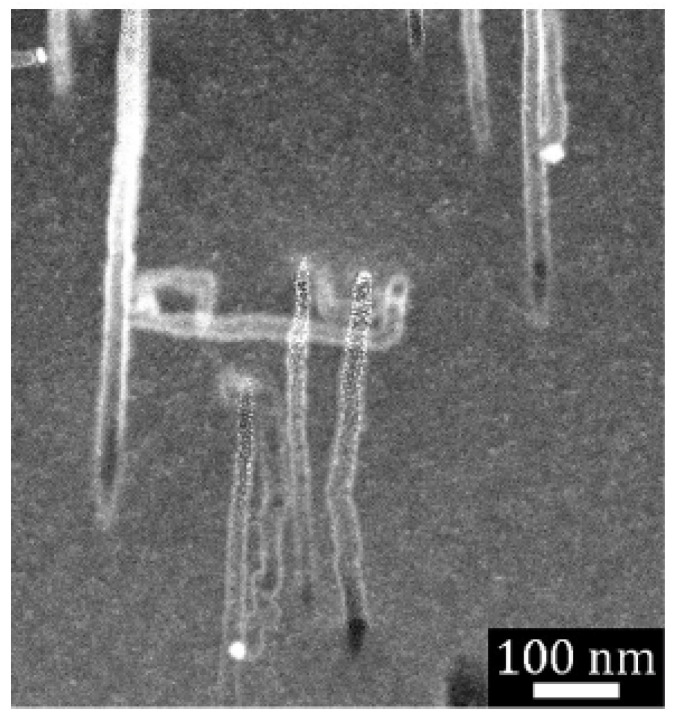
High-angle annular dark-field (HAADF) scanning transmission electron microscopy (STEM) image of focused ion beam (FIB)-cut cross section of a semiconductor grade moderately doped Si(110) wafer. Local etching results in the formation of crystallographically defined etch track pores primarily along {001} directions when HL MACE is performed. Copyright © 2021 Kolasinski, Unger, Ernst and Aindow. Reproduced under Creative Commons Attribution License (CC BY) from Ref. [72].

Cross-sectional scanning transmission electron microscopy (STEM) was used to further probe the structure of etched particles and substrates. Figure 4 displays a representative high-angle annular dark-field (HAADF) STEM image of a focused ion beam (FIB) cross section taken from a Si(110) substrate. Multiple parallel etch track pores were revealed. Several Ag nanoparticles were observed as bright spheres at the bottom of several pores. Primarily, parallel pores with a uniform length (as shown in Figure 3c) were formed, which indicated a high degree of cooperativity in the motion of metal nanoparticles during etching. Correlated motion of the high-density particles formed from high-load deposition may result from interactions derived from the overlapping space charge layers associated with each metal nanoparticle. Note that etching of substrates with moderate or low doping levels, such as the that shown in Figure 4, exhibited primarily local etching to form etch track pores of the same size as the metal nanoparticle.

**Figure 5:**
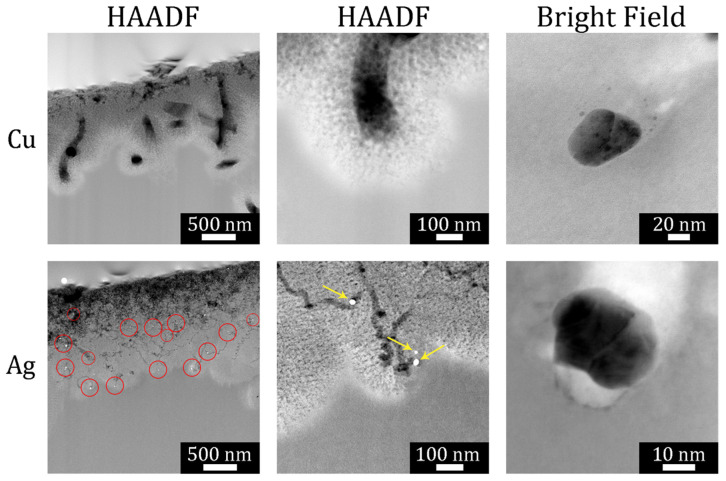
HAADF STEM image of FIB-cut cross section of a metallurgical grade Si particle etched with LL-MACE catalyzed by either Cu or Ag nanoparticles (first two columns). The bright field images in the third column show individual Cu (upper) and Ag (lower) nanoparticle catalysts embedded in Si after etching. Ag nanoparticles are observed as bright spots. Local etching results in uncorrelated, meandering etch track pores, while tortuous mesopores are created by remote etching. Reproduced with permission from Ref. [66]. Copyright © 2020, American Chemical Society.

Under low-load deposition conditions, metal nanoparticles were, on average, much further apart from one another. As shown in Figure 5, this led to uncorrelated motion of the metal nanoparticle and meandering (nonparallel) etch track pores. In addition, the etch track pores were surrounded by random, tortuous mesopores generated by remote etching.

**Figure 6:**
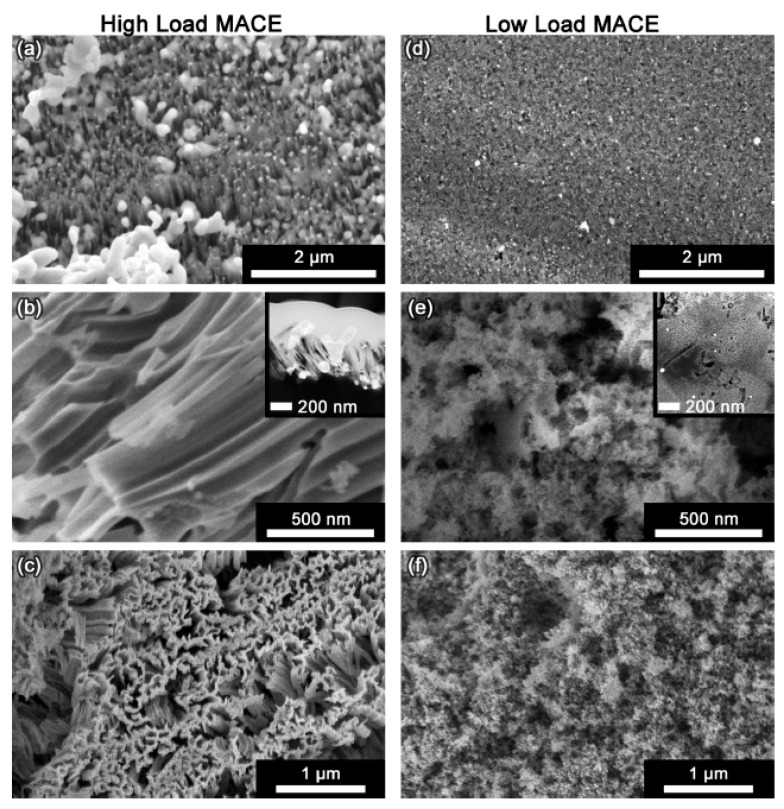
Contrasting structures associated with HL and LL regimes. High-load MACE with Ag (left-hand column) is characterized by: (**a**) Deposition of a thick metal layer composed of dendrites as well as nanoscale and microscale metal particles (plan view); (**b**) etching proceeds with correlated motion of the metal nanoparticles to form predominantly 70–100 nm parallel etch track pores (cross section); (**c**) production of etched silicon particles that are rough in texture and covered with ridges formed by the etch track pores (plan view). Low-load MACE (right-hand column) is characterized by (**d**) light deposition of dispersed individual metal nanoparticles (plan view); (**e**) uncorrelated etching of random 10–50 nm etch track pores that are surrounded by remotely etched 3–6 nm tortuous pores (cross section); (**f**) production of etched particles that are relatively smooth in texture with randomized pores (plan view). Reproduced with permission from Ref. [65]. Copyright © 2020, American Chemical Society.

The differences between HL- and LL-MACE are exhibited in Figure 6. From the structure of the deposited metal to the texture of the etched particles, the two regimes are distinctly different. Figure 6 also demonstrates that SiNWs are not formed as a direct result of etching. On the contrary, parallel etch track pores create ridges. These ridges are easily cleaved from the substrate and from each other by sonication. Efficient SiNW production is possible in this manner; however, direct formation of SiNWs only occurs when metal layers are patterned lithographically to reveal hole arrays, as first shown by Peng et al. [24] and Fang et al. [25].

**Figure 7:**
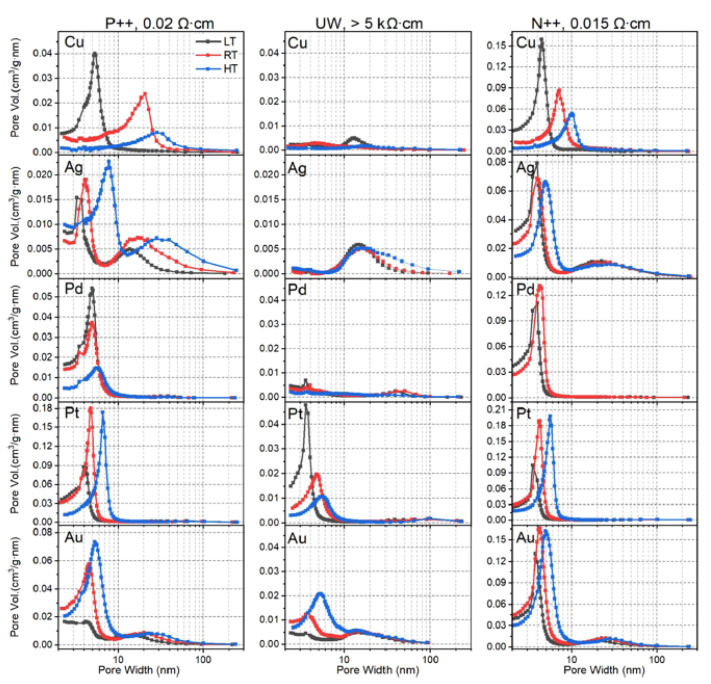
In the low-load regime (LL-MACE), control of the metal composition, doping level and temperature are used to control the pore size distribution. Reproduced with permission from Ref. [66]. Copyright © 2020, American Chemical Society.

The low-load regime is not merely a curiosity. Figure 7 demonstrates a remarkable property, i.e., LL-MACE can be used to choose the pore size distribution based upon the choice of experimental parameters. Tamarov et al. [65] found that the partitioning of remote to local etching as well as the size and behavior of the metal nanoparticle depended on (1) the elemental composition of the metal nanoparticle, (2) the doping type and level of the Si, and (3) the temperature of the etchant. By choosing between Ag, Au, Cu, Pd, and Pt over a temperature range of roughly 0–50 °C, and using different types of Si, the mean pore size was varied as well as whether the distribution was composed of one or two maxima.

The results for Cu-catalyzed MACE, shown in Figure 5 and Figure 7, demonstrate a much different type of etching than what is usually associated with Cu. The formation of black silicon has been a topic of much interest, particularly for improving the performance of photovoltaic devices [118,119,120], but also for bactericidal activity [121]. A number of groups, including [38,45,122,123,124,125,126], have generated black silicon by etching inverted pyramids into Si wafers using Cu-catalyzed MACE. These low reflectivity surfaces differentiate themselves from the better known alkaline-etched black Si [67,127] upon which upright pyramids are found. The formation of pyramidal structures in alkaline etching is understood to be related to the highly anisotropic nature of hydroxide-catalyzed hydrolysis of Si [128,129,130,131,132]. This is commonly ascribed to the necessity to form a sterically constrained transition state, with the most sterically constrained Si(111) surface exhibiting the lowest etch rate [133,134]. Analogously, anisotropic etching to reveal smooth {111} planes is involved in the formation of inverted pyramids by conventional Cu-catalyzed MACE.

Figure 5 clearly demonstrates that etching catalyzed by Cu nanoparticles does not lead to the formation of pyramidal structures. The origin of anisotropic pyramid etching may be related to the poor kinetics of Cu deposition onto Si as compared with the rather facile dissolution of Cu deposits in the presence of a sufficiently high concentration of oxidant such as H_2_O_2_. As explained above, the kinetics of Cu deposition are slow on ideal Si surfaces because the standard reduction potential of Cu is less positive than the Si VBM. It has been confirmed experimentally [4] that Cu deposition occurs initially on defect sites in contrast to Ag, Au, and Pt depositions which occur on both ideal and defect sites. Preferential deposition at step defects will lead to step-flow etching if the Cu assemblies are unable to expand onto the terraces before being dissolved. Step-flow etching is required to form the flat {111} planes observed in the inverted pyramids.

## 5. Conclusions and Perspectives

Metal-assisted catalytic etching is a micro/nano-machining technology that has been recognized for more than a decade as an extremely versatile method of processing semiconductor wafers. The ability of MACE to also process semiconductor substrates of arbitrary shape, including powders, has only been recognized much more recently. Thus, MACE is capable of producing model structures of interest to researchers, as well as functional structures useful for applications including the production of high-volume nanostructured materials. In addition, recent advances in control over the yield, pore size, and pore size distribution have demonstrated that MACE is capable of producing bespoke porosification of silicon powders in industrially relevant quantities.

## 6. Patents

One patent has been granted to the author and one application has been made based on original work reviewed here. K. W. Kolasinski and B. A. Unger, *Injection Metal Assisted Catalytic Etching*, Application No. US 62/881,636 (2019). K. W. Kolasinski, J. Salonen, and E. Mäkilä, *Regenerative Electroless Etching*, Patent No. US 10,590,562 B2 (2019).
